# Biofilm Formation, Production of Matrix Compounds and Biosorption of Copper, Nickel and Lead by Different Bacterial Strains

**DOI:** 10.3389/fmicb.2021.615113

**Published:** 2021-06-10

**Authors:** Md. Manjurul Haque, Md Khaled Mosharaf, Md. Amdadul Haque, Md. Zahid Hasan Tanvir, Md. Khairul Alam

**Affiliations:** ^1^Department of Environmental Science, Faculty of Agriculture, Bangabandhu Sheikh Mujibur Rahman Agricultural University, Gazipur, Bangladesh; ^2^Department of Agro-Processing, Faculty of Agriculture, Bangabandhu Sheikh Mujibur Rahman Agricultural University, Gazipur, Bangladesh; ^3^Soil Science Division, Bangladesh Agricultural Research Institute, Gazipur, Bangladesh

**Keywords:** biofilm, curli, cellulose, heavy metal, removal, pH, temperature, metal concentration

## Abstract

Bacterial biofilms play a key role in metal biosorption from wastewater. Recently, *Enterobacter asburiae* ENSD102, *Enterobacter ludwigii* ENSH201, *Vitreoscilla* sp. ENSG301, *Acinetobacter lwoffii* ENSG302, and *Bacillus thuringiensis* ENSW401 were shown to form air–liquid (AL) and solid–air–liquid (SAL) biofilms in a static condition at 28 and 37°C, respectively. However, how environmental and nutritional conditions affect biofilm formation; production of curli and cellulose; and biosorption of copper (Cu), nickel (Ni), and lead (Pb) by these bacteria have not been studied yet. In this study, *E. asburiae* ENSD102, *E. ludwigii* ENSH201, and *B. thuringiensis* ENSW401 developed the SAL biofilms at pH 8, while *E. asburiae* ENSD102 and *Vitreoscilla* sp. ENSG301 constructed the SAL biofilms at pH 4. However, all these strains produced AL biofilms at pH 7. In high osmolarity and ½-strength media, all these bacteria built fragile AL biofilms, while none of these strains generated the biofilms in anaerobic conditions. Congo red binding results showed that both environmental cues and bacterial strains played a vital role in curli and cellulose production. Calcofluor binding and spectrophotometric results revealed that all these bacterial strains produced significantly lesser amounts of cellulose at 37°C, pH 8, and in high osmotic conditions as compared to the regular media, at 28°C, and pH 7. Metal biosorption was drastically reduced in these bacteria at 37°C than at 28°C. Only *Vitreoscilla* sp. ENSG301 and *B. thuringiensis* ENSW401 completely removed (100%) Cu and Ni at an initial concentration of 12.5 mg l^–1^, while all these bacteria totally removed (100%) Pb at concentrations of 12.5 and 25 mg l^–1^ at pH 7 and 28°C. At an initial concentration of 100 mg l^–1^, the removal of Cu (92.5 to 97.8%) and Pb (89.3 to 98.3%) was the highest at pH 6, while it was higher (84.7 to 93.9%) for Ni at pH 7. Fourier transform infrared spectroscopy results showed metal-unloaded biomass biofilms contained amino, hydroxyl, carboxyl, carbonyl, and phosphate groups. The peak positions of these groups were shifted responding to Cu, Ni, and Pb, suggesting biosorption of metals. Thus, these bacterial strains could be utilized to remove Cu, Ni, and Pb from aquatic environment.

## Introduction

Metals at molecular densities greater than 5 g/cm^3^ are known as heavy metals (Weast, 1984). Heavy metal release from various industries [such as steel, leather, electroplating, mine tailings, paints, wastewater treatment plants, and agricultural operations (fertilizers, pesticides, and irrigations)] is one of the major causes of environmental pollution. Some heavy metals like copper (Cu), zinc (Zn), iron (Fe), cobalt (Co), chromium (Cr), and nickel (Ni) are required for growth and metabolism of organisms when they are present in trace amount, known as trace elements or micronutrients. However, they become toxic when the concentration increases. Conversely, non-essential heavy metals including lead (Pb), cadmium (Cd), mercury (Hg), and arsenic (As) are toxic even at very low concentrations. Accumulation of such heavy metals in soils and water bodies poses threat to human health (including potential carcinogenicity), other living organisms, and eventually overall biodiversity (Naser et al., 2014; Ahmed et al., 2016; Alam et al., 2017; Burakov et al., 2018). Various physicochemical technologies such as reverse osmosis, filtration, electro-dialysis, flocculation, ion exchange, activated carbon, and chemical precipitation are being practiced to remove the heavy metals from aqueous systems. However, all these methods have some disadvantages like being expensive, having high energy and reagent requirements, not being appropriate for the removal of low concentrations (1–50 mg l^–1^) of heavy metals, releasing of chemical sludge, and being less practical under natural environmental conditions (Ahluwalia and Goyal, 2007; Pan et al., 2009; Edwards and Kjellerup, 2013; Redha, 2020). Biosorption is one of the bioremediation technologies and uses fungi, bacteria, algae, and plants to sequester heavy metals (Nies, 1999). In the biosorption process, microbes adsorb metals on the cellular surface through attachment/linkage onto many anionic functional groups (Lo et al., 2014). Indeed, biosorption technique offers several benefits over the physicochemical methods in terms of economic aspects, high metal-binding capacity, eco-friendliness, and regeneration of biosorbents with the possibility of the recovery of metals (Kratochvil and Volesky, 1998; Sag and Kutsal, 2001; He and Chen, 2014; Santhosh et al., 2016). Compared to fungi, algae, and plants, bacterial strains were found effective to remove heavy metals from aquatic environment (Oves et al., 2013; Banerjee et al., 2015; Wei et al., 2016; Yang et al., 2017; El-Naggar et al., 2018). Though free-living bacterial cells have a greater capacity for metal removal from aquatic environment (Malik, 2004; Zeng et al., 2009), their survival is less likely due to decreased protection and the low metabolic activity (von Canstein et al., 2002). Hence, it is urgently needed to find out effective indigenous bacterial biosorbents that can survive even under toxic environmental conditions along with diverse metabolic states.

Bacterial biofilms are highly structured, surface-associated cells, enclosed in a matrix of self-produced extracellular polymeric substances (EPS) (Costerton et al., 1999). Compared to free-living planktonic counterparts, bacterial biofilms provide numerous benefits, including protection of cells from adverse environmental stresses (e.g., toxic chemicals, pH change, dehydration, and predation), the ability to communicate by expression of quorum-sensing molecules, exchange of genetic material (e.g., horizontal gene transfer) and nutrient availability from waste products, and persistence in different metabolic functions with respect to electron acceptor reduction (Teitzel and Parsek, 2003; Boles et al., 2004; Vu et al., 2009; Haque et al., 2012, 2017; Edwards and Kjellerup, 2013). The bacterial EPS is composed of different high-molecular-weight biopolymers including proteins, cellulose-rich polysaccharides, nucleic acids, and lipids (Flemming and Wingender, 2010; Mosharaf et al., 2018). Bacterial surface appendages including the flagella, pili, and aggregative fimbriae/curli were also reported to stabilize the biofilm matrices (Flemming et al., 2007). Several researchers have shown that negatively charged functional groups/ligands of EPS serve as a trap for heavy metal ions (Sutherland, 1984; Deng et al., 2007; Wei et al., 2016). Enzymatic activities in EPS play a key role in detoxification of heavy metals by transformation and subsequent precipitation in the polymeric mass (Pal and Paul, 2008). Both living and dead biomass biofilms can be applied to remove heavy metals from the wastewater. Among them, living biofilms were found effective to remove heavy metals from both in the continuous treatment effluents (Gadd, 2009) and in the real industrial and municipal effluents (Kotrba et al., 2011). Both biosorption and bioaccumulation simultaneously take place in living bacterial biofilms. Nevertheless, several cellular mechanisms including synthesis of specific enzymes and action of cytoplasmic or membrane proteins were shown to express in the living bacterial biofilms (Kumar et al., 2007). Therefore, instead of free-living planktonic bacteria/dead bacterial biofilms, growing (living) bacterial biofilms have been appreciated for several bacterial-dominated processes and recommended for the removal of heavy metals from the environment (Singh et al., 2006; Pal and Paul, 2008; Edwards and Kjellerup, 2013; Meliani and Bensoltane, 2016; Balan et al., 2020).

*Enterobacter asburiae* ENSD102, *Enterobacter ludwigii* ENSH201, *Vitreoscilla* sp. ENSG301, and *Acinetobacter lwoffii* are Gram-negative bacteria that are positive to catalase and acetoin tests but are negative to gelatin liquefaction, methyl red, and indole tests. Some strains of *E. asburiae* degraded polyethylene plastic (Sato et al., 2016) and augmented crop growth (Dolkar et al., 2018; Mahdi et al., 2020). *Vitreoscilla* sp. was reported to synthesize hemoglobin used in metabolic engineering (Wakabayashi et al., 1986). On the other hand, *Bacillus thuringiensis* is a Gram-positive spore-forming bacterium and well known for the production of insecticidal crystalline (Cry) proteins. Recently, all these bacterial strains were isolated from the wastewater of Bangladesh and reported to form biofilms by expression of curli (a proteinaceous component of the EPS) and nanocellulose fibers (Mosharaf et al., 2018). Important environmental applications of these bacterial strains are summarized in Table 1.

**TABLE 1:** Environmental applications of bacterial strains used in this study.

Bacteria	Important environmental applications	References
*E. asburiae*	Degradation of polythene, detoxification of carcinogenic dyes, removal of heavy metals, solubilization of nutrients, fixation of nitrogen, crop growth promoter, utilization as biofertilizer, and reduction of metal toxicity in crop plants.	Jetiyanon, 2015; Kang et al., 2015; Paul and Mukherjee, 2016; Ren et al., 2019; Mahdi et al., 2020; Haque et al., 2021a
*E. ludwigii*	Degradation of dyes, biosorption of heavy metals, functions as nematicides, solubilization of nutrients, reduction of cadmium stress in plants, plant growth enhancer, and prevention of drought and salinity stress in crop plants.	Shoebitz et al., 2009; Gontia−Mishra et al., 2016; Radwan et al., 2017; Adhikari et al., 2020; Haque et al., 2021b
*Vitreoscilla* sp.	Decolorization, degradation, and detoxification of textile dyes; hemoglobin technology used in bio-product synthesis; bioremediation; and enhancement of tolerance of submergence, oxidative, and nitrosative stress in plants.	Stark et al., 2015; Zelasco et al., 2006; Haque et al., 2021a, b
*A. lwoffii*	Detoxification of dyes, biodegradation of diesel, bioremediation of heavy metals, and solubilization of phosphate.	Sadiq et al., 2013; Mindlin et al., 2016; Imron and Tttah, 2018; Haque et al., 2021a, b
*B. thuringiensis*	Decolorization of azo dyes; degradation of naproxen, ibuprofen, and chlorpyrifos; removal of heavy metals; used in insect control; plant growth activator; solubilization of nutrients; fixation of nitrogen; and mitigation of drought stress in plants.	Oves et al., 2013; Armada et al., 2014; Aceves-Diez et al., 2015; de Maagd, 2015; Marchlewicz et al., 2016; Martins et al., 2018; Haque et al., 2021a, b

Several studies have shown that environmental conditions affect biofilm formation (Rinaudi et al., 2006; Liang et al., 2010; Haque et al., 2015; Ross et al., 2018) and the expression of curli and cellulose in different bacterial strains (Gerstel and Römling, 2003; Barnhart and Chapman, 2006). Initial metal concentration, temperature, pH, and contact time were shown to affect the biosorption of heavy metals (Oves et al., 2013; Yang et al., 2017; El-Naggar et al., 2018; Redha, 2020). Among the factors, pH plays a key role in the metal speciation, metal solubility, and dissociation of functional groups present in the bacterial cell wall (Esposito et al., 2002). Metal ions in solution undergo hydrolysis as the pH increases. However, the extent of hydrolysis at different pH values differs with each metal, but the usual sequence of hydrolysis is the formation of hydroxylated monomeric species followed by the formation of polymeric species and then the formation of crystalline oxide precipitates after aging (Bacs and Mesmer, 1976). Therefore, adsorption of metals on interfaces is highly pH-dependent. For example, Cu can be present in solution as three different species: Cu^2+^, CuOH^+^, and Cu(OH)_2_. Cu^2+^ and CuOH^+^ are more favorable Cu species under lower pH conditions (Yang et al., 2017). Cu, Ni, and Pb are frequently found in industrial wastewater, rivers, sediments, fish, and vegetables in Bangladesh (Naser et al., 2014; Ahmed et al., 2016; Mosharaf et al., 2018; Uddin and Jeong, 2021). Concentrations of these metals were also reported to increase day by day in the environment of Bangladesh (Uddin and Jeong, 2021). Thus, it is urgently needed to study the biosorption of Cu, Ni, and Pb from the environment. How environmental factors affect biofilm formation, the expression of biofilm matrix components (e.g., curli and cellulose), and the biosorption of Cu, Ni, and Pb has never been investigated in *E. asburiae* ENSD102, *E. ludwigii* ENSH201, *Vitreoscilla* sp. ENSG301, *A. lwoffii* ENSG302, and *B. thuringiensis* ENSW401. Therefore, it is aimed to quantify the effects of different environmental cues such as temperature, pH, osmolarity, oxygen tension, and nutritional strength on biofilm formation and production of curli and cellulose in these bacterial strains. It is also intended to evaluate these bacterial strains for their efficacies to remove Cu, Ni, and Pb from aqueous solutions in response to initial metal concentration, temperature, and pH. Furthermore, it is aimed to identify the chemical functional groups/ligands present in both metal-unloaded and metal-loaded biomass biofilms produced by these bacterial strains using Fourier transform infrared (FTIR) spectroscopy. The study will contribute toward understanding the mechanisms and potential of these bacterial strains in biosorption of heavy metals from aquatic environment.

## Materials and Methods

### Chemicals

Heavy metals {copper sulfate (CuSO_4_.5H_2_O), nickel sulfate (NiSO_4_.6H_2_O), and lead nitrate [Pb(NO_3_)_2_]}, Congo red, and Calcofluor were obtained from Sigma-Aldrich (St. Louis, MO, United States). All other chemicals used were of an analytical grade and were purchased from Wako Pure Chemical Industries, Ltd. (Osaka, Japan), Bio Basic Canada Inc. (Markham, ON, Canada), and HiMedia (Mumbai, India).

### Bacterial Strains and Growth Conditions

*Enterobacter asburiae* ENSD102, *E. ludwigii* ENSH201, *Vitreoscilla* sp. ENSG301, *A. lwoffii* ENSG302, and *B. thuringiensis* ENSW401 used in this study were recently isolated from wastewaters of Bangladesh and reported to form air–liquid (AL) and solid–air–liquid (SAL) biofilms on salt-optimized broth plus glycerol [(SOBG, pH 7.0) (per liter, 20 g tryptone, 5 g yeast extract, 0.5 g NaCl, 2.4 g MgSO4, 0.186 g KCl, and 2% glycerol] in a static condition at 28 and 37°C, respectively (Mosharaf et al., 2018). In this study, all these bacterial strains were found non-pathogenic to human and animals based on hemolytic test performed using 5% sheep blood (data not shown). These strains were routinely grown in yeast extract peptone (YP) medium (pH 7) at 28°C. An absorption spectrophotometer (Intertech, Inc., Tokyo, Japan) was used to measure the bacterial optical density (OD) at 660 nm.

### Preparation of Inoculum for Different Experiments

In order to prepare inoculum for different studies, a single colony of each bacterium was inoculated in YP broth and incubated at 28°C in a shaking (160 rpm) condition until OD_660_ reached at 0.6–0.8. Then, each bacterial culture was harvested and centrifuged at 10,000 rpm for 5 min. The supernatant was carefully discarded and the pellet was re-suspended in YP broth and used for the experiments.

### Role of Environmental Factors on Biofilm Formation and Expression of Curli and Cellulose

To quantify the effect of pH, pH of the SOBG was adjusted to pH 4, 7, and 8 by adding malic acid or NaOH and autoclaved. For biofilm formation, a 50-μl culture [ca. 10^5^ colony forming unit (CFU) ml^–1^] of each bacterium was suspended in the glass test tubes (Pyrex, flat bottom) containing 5 ml SOBG at pH 4, 7, or 8. For high osmolarity, SOBG (pH 7) was supplemented with 0.3 M NaCl or 0.3 M D-sorbitol. To examine the role of oxygen tension on biofilm formation, a 50-μl culture of each strain was inoculated in the glass test tubes containing 5 ml SOBG broth and then sealed with paraffin oil (1.5 ml) or without sealing with paraffin oil. The inoculated test tubes were incubated in an incubator or in an anaerobic chamber (Thermo Fisher Scientific, Inc., Portsmouth, NH, United States) at 28°C in a static condition. To study the effect of nutritional strength on biofilm formation, ½-strength SOBG (pH 7) was used instead of full strength. In all the cases, AL or SAL biofilm was identified within 72-h incubation as described in Haque et al. (2012) and photographs were taken. All the biofilm formation assays were performed three times.

The role of environmental cues [temperature (28 and 37°C), pH (4, 7, and 8), high osmolarity, and nutritional strength] on the expression of curli and cellulose in different bacterial strains was also studied. Congo red binding assays were performed to detect both curli and cellulose production as described by Haque et al. (2017). Calcofluor binding assays were also done to detect specifically the cellulose production as described by Haque et al. (2017). Moreover, cellulose production by different bacterial strains in response to different environmental cues was quantified by using a spectrophotometer (Ultrospec-3000, Pharmacia Biotech, Cambridge, England) as described by Haque et al. (2017). All these assays were performed twice.

### Determination of Maximum Tolerance Concentration of Cu, Ni, and Pb

The maximum tolerance concentration (MTC) was determined as the method described in Schmidt and Schlegel (1994) with a few modifications. In brief, a single colony of each bacterial strain was inoculated in the glass test tube containing 5 ml YP broth and incubated at 28°C in a shaking condition until optical density (OD_660_) reached at 1.0. Then, 50 μl (ca. 10^7^ CFU ml^–1^) culture of each strain was spread onto YP agar plates (three plates/concentration) containing 0 to 1,000 mg l^–1^ Cu, Ni, or Pb and incubated at 28°C in a static condition. The MTC was designated as the highest concentration of Cu, Ni, and Pb that allows confluent growth after 72 h of incubation. This experiment was repeated three times.

### Initial Metal Concentration, Temperature, and pH on Biosorption of Cu, Ni, and Pb

To examine the effect of initial heavy metal ion concentration on biosorption, a 50-μl suspension (ca. 10^6^ CFU ml^–1^) of each bacterium was inoculated in 5 ml SOBG (pH 7) supplemented with 12.5, 25, 50, 100, 150, or 200 mg l^–1^ Cu, Ni, or Pb. The inoculated test tubes were incubated at 28°C in a static condition. To evaluate the impacts of pH, the pH of the SOBG containing 100 mg l^–1^ Cu, Ni, or Pb was adjusted to pH 5, 6, 7, 8, and 9 and then autoclaved. The inoculation was done as described above and then kept at 28°C in a static condition. To quantify the effect of temperature, a 50-μl suspension (ca. 10^6^ CFU ml^–1^) of each strain was inoculated in SOBG (pH 7) with 100 mg l^–1^ Cu, Ni, or Pb and then incubated at two different temperatures such as 28 and 37°C in a static condition. In all the cases, 1-ml culture was collected from beneath of the biofilms after 72-h incubation and centrifuged at 15,000 rpm for 15 min. Heavy metals such as Cu, Ni, or Pb from the supernatants were determined using an atomic absorption spectrophotometer (VARIAN model AA2407). All these experiments were repeated twice. The percentage of metal removal efficiency (% RE) was calculated using the following equation:

$$\%\mathrm{RE}=\frac{\left(\mathrm{Co}-\mathrm{Cf}\right)}{\text{Co}}\times 100$$

where Co and Cf are initial and final concentrations (mg l^–1^) of Cu, Ni, or Pb in the solution, respectively.

### Identification of Functional Groups in Metal-Loaded and -Unloaded Biomass Biofilms Using FTIR Spectroscopy

Initially, a 50-μl suspension (ca. 10^6^ CFU ml^–1^) of each bacterium was inoculated in glass test tubes containing 5 ml SOBG (pH 7) or 5 ml magnesium-deprived SOBG (pH 7) containing 100 mg l^–1^ Cu, Ni, or Pb. Then, the tubes were incubated at 28°C in a static condition. After 72-h incubation, each bacterial biofilm was carefully collected and centrifuged at 14,000 rpm for 10 min. Each pellet was scanned (450 to 4,000 cm^–1^, 16 scans at 4 cm^–1^ resolution) at 0.2 cm s^–1^ scanning speed using a triglycine sulfate (TGS) detector. The IR spectra of the metal-unloaded and metal-loaded biofilm matrices were acquired using the PerkinElmer FTIR spectroscopy (Spectrum-2) instrument operated by CPU32M software. PerkinElmer’s proprietary software (version 10.05.03) was used to analyze the baseline-subtracted biofilm spectra.

### Experimental Design and Statistical Analysis

All the experiments were performed in a complete randomized design with at least three replications and repeated at least three times unless otherwise stated. Analysis of variance (ANOVA), distribution of data, homogeneity of variance, and mean comparison were analyzed using the R software version 3.3.6. The Fisher’s least significant difference test was applied to compare the means.

## Results

### Effect of pH on Biofilm Formation

**FIGURE 1:**
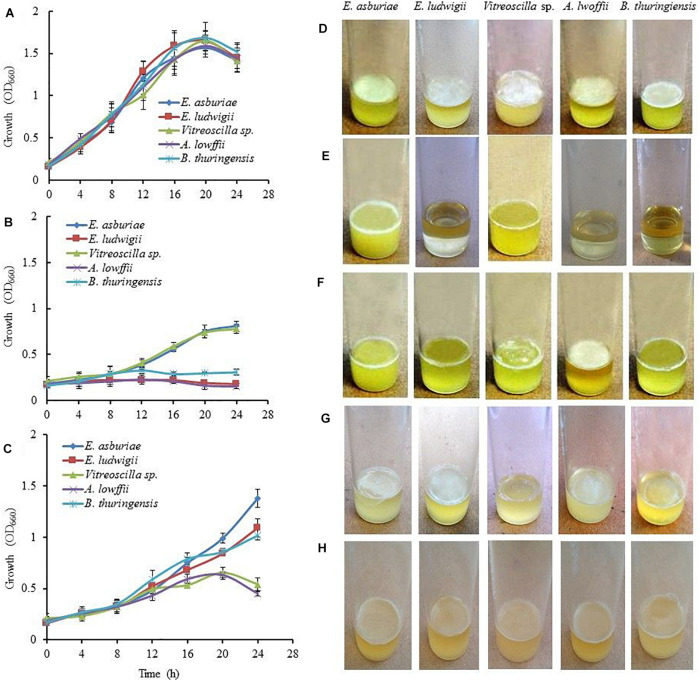
The Effect of environmental cues on bacterial growth and biofilm formation. Growth of the bacterial strains in SOBG at pH 7 **(A)**, pH 4 **(B)**, and high osmotic condition, i.e., SOBG containing 0.3 M NaCl **(C)** in a shaking condition. Biofilm formation on SOBG at pH 7 **(D)**, pH 4 **(E)**, pH 8 **(F)**, SOBG containing 0.3 M NaCl **(G)**, and ½-strength SOBG **(H)** by different bacterial strains after 72-h incubation in a static condition. The values are mean, and error bars indicate standard deviations (±) of three independent experiments. SOBG, salt-optimized broth plus glycerol.

Information regarding the role of pH on biofilm formation by *E. asburiae* ENSD102, *E. ludwigii* ENSH201, *Vitreoscilla* sp. ENSG301, *A. lwoffii* ENSG302, and *B. thuringiensis* ENSW401 were not available in the literature. Therefore, these bacterial strains were evaluated for their abilities to form biofilm in response to different pH levels. Initially, growth of these strains was examined in SOBG media at pH 4, 7, and 8 under a shaking condition (160 rpm) at 28°C. All these strains grew rapidly both at pH 7 (Figure 1A) and pH 8 (data not shown). However, the growth rate of these strains was not significantly differed at pH 7 and pH 8 (data not shown). At pH 4, *E. ludwigii* ENSH201, *A. lwoffii* ENSG302, and *B. thuringiensis* ENSW401 were unable to grow (Figure 1B), while *E. asburiae* ENSD102 (OD_660_ = 0.81 at 24 h) and *Vitreoscilla* sp. ENSG301 (OD_660_ = 0.78 at 24 h) cells grew moderately (Figure 1B). Like growth, biofilm formation was also affected by different pH levels. All these bacterial strains confirmed the development of thick and robust AL biofilms at pH 7 (Figure 1D). *E. asburiae* ENSD102 and *Vitreoscilla* sp. ENSG301 produced a prominent SAL biofilms at pH 4 (Figure 1E). *A. lwoffii* ENSG302 built a strong AL biofilm at pH 8, while *Vitreoscilla* sp. ENSG301 created a weak and fragile AL biofilm at pH 8 (Figure 1F). Conversely, *E. asburiae* ENSD102, *E. ludwigii* ENSH201, and *B. thuringiensis* ENSW401 developed the SAL biofilms at pH 8 (Figure 1F). Thus, these bacterial strains synthesized two types of biofilms, i.e., AL and SAL biofilm, depending on the pH levels.

### The Effect of Osmolarity and Availability of Oxygen on Biofilm Formation

When these bacterial strains were grown in SOBG (pH 7) supplemented with 0.3 M NaCl in a shaking condition at 28°C, the growth rate of *Vitreoscilla* sp. ENS301 and *A. lwoffii* ENSG302 was drastically reduced as compared to that of *E. asburiae* ENSD102, *E. ludwigii* ENSH201, and *B. thuringiensis* ENSW401 (Figure 1C). Similar results were also found when SOBG (pH 7) was supplemented with 0.3 M D-sorbitol (data not shown). Like bacterial growth, biofilm formation in all these strains was dramatically reduced in high-osmolarity media (Figure 1G) as compared to that of regular SOBG media (Figure 1D). Taken together, these observations suggest that both NaCl and D-sorbitol negatively affect biofilm formation through an osmotic effect.

None of these strains formed the biofilm in the glass test tubes containing 5 ml SOBG broth sealed with paraffin oil after 72-h incubation at 28°C in a static condition (data not shown). Similar results were also found when inoculated tubes were placed in an anaerobic chamber at 28°C (data not shown).

### The Effect of the Nutritional Conditions on Biofilm Formation

Bacterial growth was indistinguishable in ½-strength SOBG (data not shown) and full-strength SOBG. However, all these bacterial strains formed a lighter and fragile AL biofilms in ½-strength SOBG after 72-h incubation at 28°C in a static condition (Figure 1H). These bacterial strains did not build AL or SAL biofilm in magnesium-deprived SOBG media (Mosharaf et al., 2018). When magnesium-deprived SOBG media was supplemented with 0.009 M Ca^2+^, Cu^2+^, and Zn^2+^, all these strains developed dense and stout AL biofilms (data not shown). Thus, media composition plays an important role in biofilm formation of these bacteria.

### Effect of Initial Cu, Ni, and Pb Concentrations on Biomass Biofilm Formation

Biofilm formation in terms of the production of biomass (wet) biofilms by *E. asburiae* ENSD102, *E. ludwigii* ENSH201, *Vitreoscilla* sp. ENSG301, *A. lwoffii* ENSG302, and *B. thuringiensis* ENSW401 was also examined in response to different concentrations (12.5, 25, 50, 100, 150, and 200 mg l^–1^) of Cu, Ni, and Pb. Biomass biofilm production (wet) was not significantly differed in these bacterial strains responding to 12.5 to 200 mg l^–1^ Cu, Ni, and Pb (Supplementary Figure 1). However, biomass biofilm production (mean) was higher in different concentrations of Cu (11.45 mg l^–1^) and Ni (11.53 mg l^–1^) as compared to the varying concentrations of Pb (9.52 mg l^–1^) in these bacterial strains. The amount of biofilm production by *E. asburiae* ENSD102, *E. ludwigii* ENSH201, *Vitreoscilla* sp. ENSG301, *A. lwoffii* ENSG302, and *B. thuringiensis* ENSW401 ranged from 11.2 to 11.5, 11.2 to 11.6, 11.3 to 11.6, 11.3 to 11.6, and 11.4 to 11.7 mg ml^–1^, respectively, in response to 12.5 to 200 mg l^–1^ Cu (Supplementary Figure 1A). In case of Ni, it varied from 11.4 to 11.7, 11.1 to 11.5, 11.5 to 11.7, 11.4 to 11.6, and 11.5 to 11.8 mg ml^–1^ in *E. asburiae* ENSD102, *E. ludwigii* ENSH201, *Vitreoscilla* sp. ENSG301, *A. lwoffii* ENSG302, and *B. thuringiensis* ENSW401, respectively, responding to 12.5 to 200 mg l^–1^ (Supplementary Figure 1B). In 12.5 to 200 mg l^–1^ Pb, production of biomass biofilms by *E. asburiae* ENSD102, *E. ludwigii* ENSH201, *Vitreoscilla* sp. ENSG301, *A. lwoffii* ENSG302, and *B. thuringiensis* ENSW401 fluctuated from 9.3 to 9.7, 9.2 to 9.7, 9.5 to 9.9, 9.2 to 9.5, and 9.5 to 9.7 mg ml^–1^, respectively (Supplementary Figure 1C).

### Congo Red Binding Assays

**FIGURE 2:**
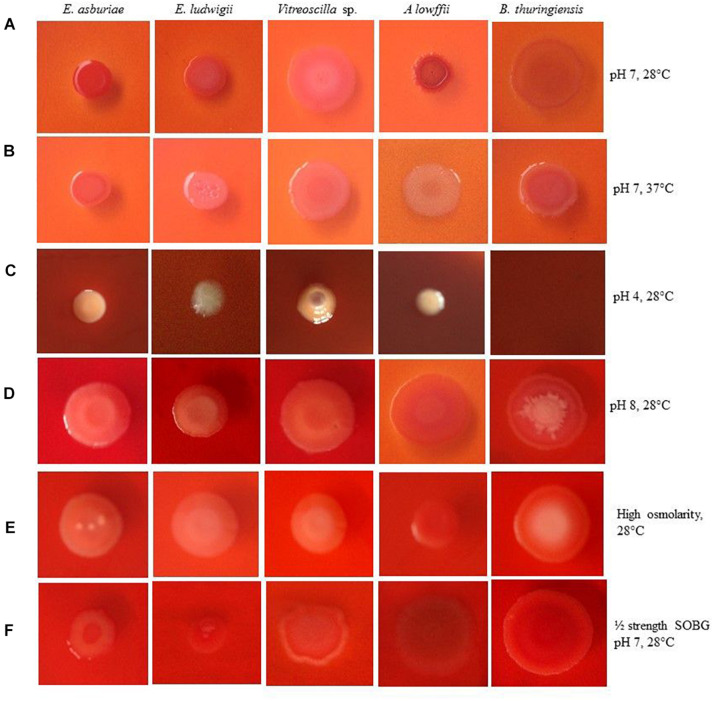
Congo red binding abilities of different bacterial strains in response to pH 7 at 28°C **(A)**, pH 7 at 37°C **(B)**, pH 4 at 28°C **(C)**, pH 8 at 28°C **(D)**, high osmolarity at 28°C **(E)** and ½ strength SOBG, pH 7 at 28°C **(F)** after 48-h incubation. Photographs represent one of two experiments, which gave similar results.

Numerous studies have shown that biofilm-producing bacteria expressed curli and cellulose – two important components of the biofilm matrices (Römling, 2005; Haque et al., 2009, 2012, 2017; Milanov et al., 2015). Milanov et al. (2015) have demonstrated that bacterial strains producing the red, dry, and rough (rdar) phenotype on Congo red agar plates leads to both curli and cellulose, while only cellulose expresses the pink, dry, and rough (pdar) phenotype and only curli induces the brown, dry, and rough (bdar) phenotype. When no components are expressed, the phenotype is smooth and white (saw). Bokranz et al. (2005), on the other hand, have reported that bacterial strains generating the red and smooth (ras) and pink and smooth (pas) lead to only cellulose, while brown and smooth (bas) triggers only curli in certain *E. coli* strains. However, the effect of temperature, pH, osmolarity, and nutritional strength on the expression of curli and cellulose has never been investigated in *E. asburiae* ENSD102, *E. ludwigii* ENSH201, *Vitreoscilla* sp. ENSG301, *A. lwoffii* ENSG302, and *B. thuringiensis* ENSW401 by any other researches. Thus, the phenotypes were examined in response to different environmental conditions, and the results are depicted in Figure 2.

*Enterobacter asburiae* ENSD102 and *B. thuringiensis* ENSW401 expressed the rdar phenotypes, linked with both curli and cellulose production at both temperatures, i.e., 28 and 37°C (Figures 2A,B), while *Vitreoscilla* sp. ENSG301 triggered the pdar phenotypes, associated with cellulose only at both temperatures (Figures 2A,B). *E. ludwigii* ENSH201 and *A. lwoffii* ENSG302 produced the rdar and pdar phenotypes at 28°C (Figure 2A) and 37°C (Figure 2B), respectively.

When pH of the SOBG Congo red broth was adjusted to 4 with malic acid, the color of the SOBG media was slightly changed after being autoclaved (Figure 2C). At pH 4, *E. asburiae* ENSD102 and *Vitreoscilla* sp. ENSG301 induced the bdar phenotype (Figure 2C), linked with curli production only, while *E. ludwigii* ENSH201 and *A. lwoffii* ENSG302 produced the saw (no expression of cellulose or curli) phenotypes (Figure 2C). At pH 8, *E. asburiae* ENSD102, *E. ludwigii* ENSH201, and *Vitreoscilla* sp. ENSG301 triggered the pas phenotypes (Figure 2D), while *A. lwoffii* ENSG302 produced the rdar phenotype (Figure 2D). Furthermore, the center of the colonies of *B. thuringiensis* ENSW401 was decorated and pink in color, while the side of the colonies was red at pH 8 (Figure 2D).

Like pH 8, *E. asburiae* ENSD102, *E. ludwigii* ENSH201, and *Vitreoscilla* sp. ENSG301 also constructed the pas phenotypes in high osmotic condition (Figure 2E), while *A. lwoffii* ENSG302 developed the rdar phenotype at the same condition (Figure 2E). In *B. thuringiensis* ENSW401, the side of the colony was red and the center was pink in color but not decorated like pH 8 (Figure 2E). All these bacterial strains developed the rdar phenotypes in ½-strength SOBG. Thus, both environmental cues and bacterial strains might be involved in the production of curli and cellulose.

### Calcofluor Binding Assays

**FIGURE 3:**
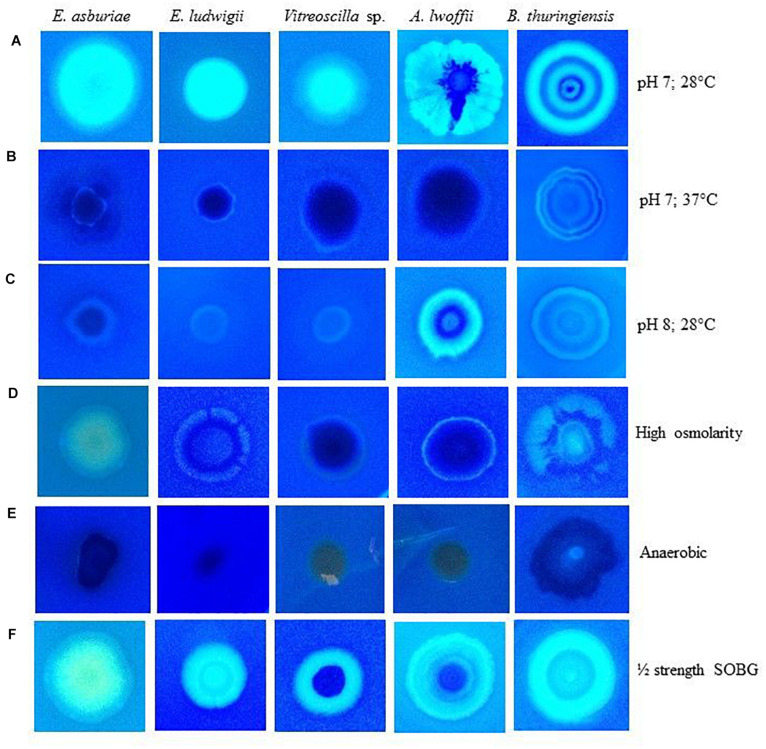
Calcofluor binding abilities of different bacterial strains in response to pH 7 at 28°C **(A)**, pH 7 at 37°C **(B)**, pH 8 at 28°C **(C)**, high osmolarity at 28°C **(D)**, anaerobic condition **(E)** and ½ strength SOBG at 28°C **(F)** after 48-h incubation. Photographs represent one of two experiments, which gave similar results.

The rdar/pdar/pas-expressing bacteria were also shown to bind with Calcofluor, a cellulose-specific dye (Römling, 2005; Steenackers et al., 2012; Haque et al., 2017). The abilities of these bacteria to bind with Calcofluor were also found to be influenced by various environmental factors (Figure 3). All these bacterial strains fluoresced strongly at 28°C and at pH 7 (Figure 3A), while they fluoresced only slightly at 37°C (Figure 3B) and at pH 7. At pH 8, except *A. lwoffii* ENSG302, all the other strains fluoresced weakly (Figure 3C). All these bacterial strains also weakly fluoresced in high osmotic condition (Figure 3D). As expected, no fluorescence was observed when these strains were incubated in an anaerobic chamber (Figure 3E). Interestingly, all these strains fluoresced strongly in ½-strength SOBG (Figure 3F). Thus, inability/weak/fragile biofilm formation by these strains might be due to no/lesser amounts of cellulose production.

### Spectrophotometric Determination of Cellulose

**FIGURE 4:**
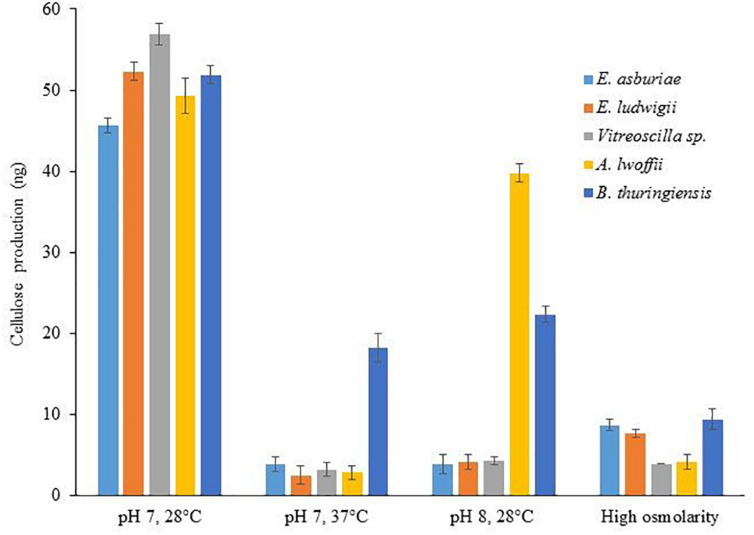
Cellulose production by different biofilm-producing bacterial strains in different environmental conditions. Cellulose was isolated from 250 mg of lyophilized cell mass obtained from each bacterium grown on Calcofluor agar plates after 48-h incubation at 28°C. The amount of cellulose was determined (OD_620_) by addition of anthrone reagent, and Avicel cellulose was used as standard. The values are mean, and error bars indicate standard deviations (±) of two independent experiments.

Differences of Calcofluor binding were shown through the discrepancy of cellulose production in bacteria (Anriany et al., 2006; Haque et al., 2017). Therefore, cellulose production was quantified in different bacterial strains grown in various environmental conditions (Figure 4). In this study, cellulose production was reduced by 12. 0-, 20. 9-, 17. 7-, 17. 6-, and 2.8-fold in *E. asburiae* ENSD102, *E. ludwigii* ENSH201, *Vitreoscilla* sp. ENSG301, *A. lwoffii* ENSG302, and *B. thuringiensis* ENSW401, respectively, at 37°C as compared at 28°C. Cellulose production was also reduced in these strains responding to pH 8 (than at pH 7) and high osmolarity (compared to regular SOBG). Thus, increase of fluorescence in Calcofluor agar plates seemed to have been reflected in the increase of cellulose production in these bacteria.

### Maximum Tolerance Concentration of Cu, Ni, and Pb by Different Bacterial Strains

Before biosorption studies, the MTC of different heavy metals including Cu, Ni, and Pb by *E. asburiae* ENSD102, *E. ludwigii* ENSH201, *Vitreoscilla* sp. ENSG301, *A. lwoffii* ENSG302, and *B. thuringiensis* ENSW401 was determined. The MTC of Cu, Ni, and Pb was remarkably varied in these bacterial strains (Table 2). All these bacterial strains showed the highest (500 to 650 mg l^–1^) MTC in response to Pb. The MTC of Cu ranged from 250 to 600 mg l^–1^. Among the bacterial strains, *B. thuringiensis* ENSW401 exhibited the highest MTC (600 mg l^–1^) in response to Cu followed by *Vitreoscilla* sp. ENSG301 (550 mg l^–1^), *A. lwoffii* ENSG302 (400 mg l^–1^), *E. ludwigii* ENSH201 (300 mg l^–1^), and *E. asburiae* ENSD102 (250 mg l^–1^). However, the MTC of Ni by these bacterial strains was fluctuated from 200 to 300 mg l^–1^. Thus, the MTC might be dependent on both metals and bacterial strains used in the study.

**TABLE 2:** Maximum tolerance concentration (MTC) of heavy metals by different bacterial strains.

Bacterial strains	MTC of different heavy metals (mg l^–1^)		
	Cu	Ni	Pb
*E. asburiae* ENSD102	250	250	500
*E. ludwigii* ENSH201	300	200	500
*Vitreoscilla* sp. ENSG301	550	250	550
*A. lwoffii* ENSG302	400	200	550
*B. thuringiensis* ENSW401	600	300	650

### The Effect of Initial Metal Concentration on the Biosorption of Cu, Ni, and Pb

**FIGURE 5:**
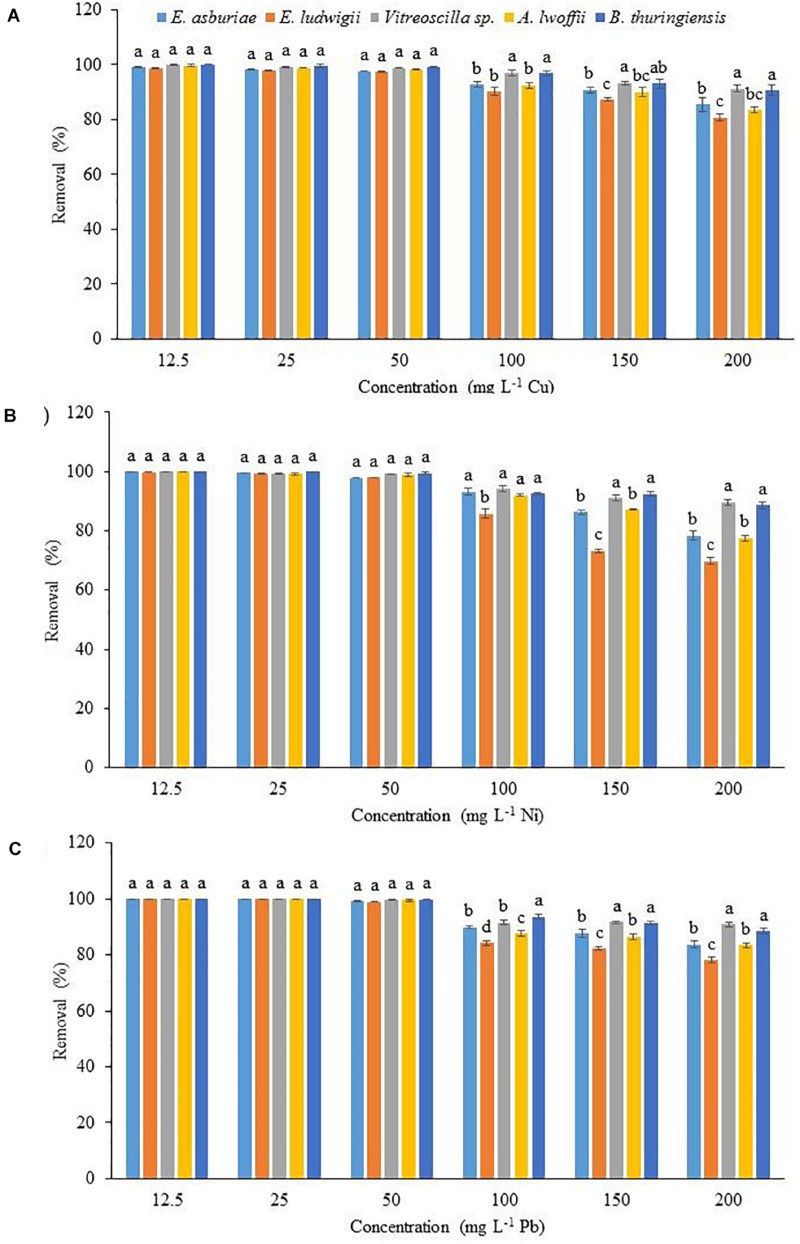
The effect of initial metal concentration on the biosorption of Cu **(A)**, Ni **(B)**, and Pb **(C)** by different bacterial strains at pH 7 and 28°C after 72 h. The values are mean, and error bars indicate standard deviations (±) of two independent experiments.

The physicochemical technologies are not suitable for the removal of low concentrations (1 to 50 mg l^–1^) of heavy metals from wastewater. Therefore, the effect of initial concentrations of 12.5, 25, 50, 100, 150, and 200 mg l^–1^ Cu, Ni, and Pb on metal uptake by *E. asburiae* ENSD102, *E. ludwigii* ENSH201, *Vitreoscilla* sp. ENSG301, *A. lwoffii* ENSG302, and *B. thuringiensis* ENSW401 was examined. The biosorption rate was not significantly differed in these bacterial strains at initial concentrations of 12.5, 25, and 50 mg l^–1^ Cu, Ni, and Pb, respectively (Figure 5). Surprisingly, *Vitreoscilla* sp. ENSG301 and *B. thuringiensis* ENSW401 completely removed (100%) both Cu (Figure 5A) and Ni (Figure 5B) at an initial concentration of 12.5 mg l^–1^. On the other hand, all these bacterial strains completely removed (100%) Pb at initial concentrations of 12.5 and 25 mg l^–1^ (Figure 5C). At an initial concentration of 50 mg l^–1^, the removal percentage of Cu ranged from 97.8 to 99.4% by these bacterial strains (Figure 5A). Ni and Pb ranged from 98.9 to 99.8% and 99.1 to 99.8%, respectively, by these bacterial strains (Figure 5C). *Vitreoscilla* sp. ENSG301 sorped the highest percentage (97.1%) of Cu at an initial concentration of 100 mg l^–1^, which was statistically akin with *B. thuringiensis* ENSW401 (96.8%). *E. asburiae* ENSD102, *E. ludwigii* ENSH201, and *A. lwoffii* ENSG302 removed 92.7, 90.2, and 92.5% Cu, respectively, which were incredibly differed from *Vitreoscilla* sp. ENSG301 and *B. thuringiensis* ENSW401 at an initial concentration of 100 mg l^–1^ Cu (Figure 5A). The biosorption percentage of Ni by *Vitreoscilla* sp. ENSG301 was also significantly higher (94.2%) at an initial concentration of 100 mg l^–1^, which was statistically alike with *E. asburiae* ENSD102, *A. lwoffii* ENSG302, and *B. thuringiensis* ENSW401 (Figure 5B). On the other hand, *B. thuringiensis* ENSW401 removed significantly the maximum Pb (93.5%) followed by *Vitreoscilla* sp. ENSG301 (91.5%), *E. asburiae* ENSD102 (89.8%), *A. lwoffii* ENSG302 (87.6%), and *E. ludwigii* ENSH201 (84.2%) at an initial concentration of 100 mg l^–1^ (Figure 5C). A significant percentage of Cu (80.7 to 91.2%), Ni (69.8 to 89.6%), and Pb (78.1 to 88.5%) was also removed by these bacterial strains at an initial concentration of 200 mg l^–1^ (Figure 5).

### The Effect of Temperature and pH on the Biosorption of Cu, Ni, and Pb

**FIGURE 6:**
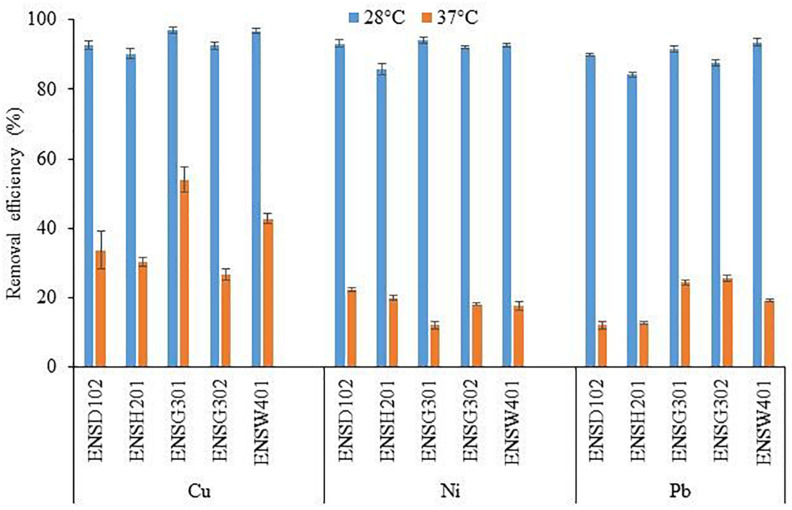
The effect of different temperatures on the biosorption of Cu, Ni, and Pb by different bacterial strains at an initial concentration of 100 mg l^–1^ and pH 7 after 72 h. The values are mean, and error bars indicate standard deviations (±) of two independent experiments.

The biosorption of Cu, Ni, and Pb by different bacterial strains was significantly influenced by temperature as shown in Figure 6. Copper biosorption was reduced by 2. 76-, 2. 98-, 1. 80-, 3. 48-, and 2.26-fold in *E. asburiae* ENSD102, *E. ludwigii* ENSH201, *Vitreoscilla* sp. ENSG301, *A. lwoffii* ENSG302, and *B. thuringiensis* ENSW401, respectively, at 37°C as compared with 28°C, while Ni biosorption was diminished by 4. 17-, 4. 29-, 7. 85-, 5. 08-, and 5.32-fold in these bacterial strains. Lead biosorption by *E. asburiae* ENSD102, *E. ludwigii* ENSH201, *Vitreoscilla* sp. ENSG301, *A. lwoffii* ENSG302, and *B. thuringiensis* ENSW401 at 37°C was also decreased by 7. 42-, 6. 68-, 3.75, 3. 43-, and 4.9-fold than at 28°C. Thus, temperature plays a crucial role in the biosorption of Cu, Ni, and Pb.

**FIGURE 7:**
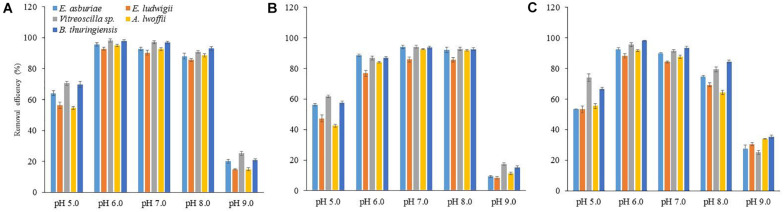
The effect of pH on the biosorption of Cu **(A)**, Ni **(B)**, and Pb **(C)** by different bacterial strains at an initial concentration of 100 mg l^–1^ for each metal at 28°C after 72 h. The values are mean, and error bars indicate standard deviations (±) of two independent experiments.

To study the impact of pH on metal biosorption, an experiment was set up with varying pH (5, 6, 7, 8, and 9) with 100 mg l^–1^ of Cu, Ni, or Pb (Figure 7). The abilities of biosorption for each metal ion by different bacterial strains increased with an increase in pH up to 8, while biosorption capacities of these metal ions decreased at pH 9. The Cu and Pb biosorption was the highest at pH 6, while it was higher for Ni at pH 7 than the sorption at other pH.

### FTIR Analysis

**FIGURE 8:**
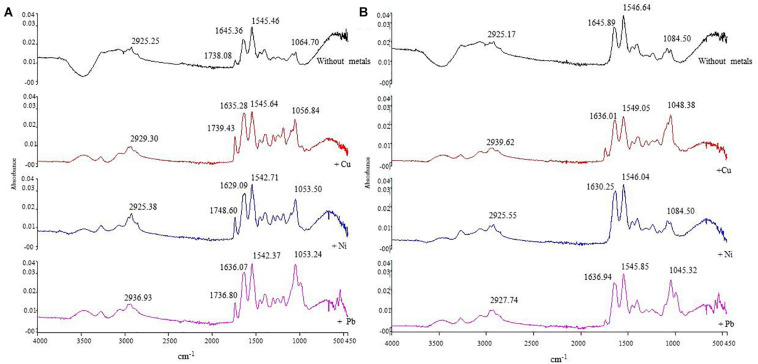
The IR spectra of metal-unloaded and metal-loaded (100 mg l^–1^ of Cu, Ni, or Pb) biomass biofilms of *Vitreoscilla* sp. ENSG301 **(A)** and *B. thuringiensis* ENSW401 **(B)** at pH 7 and at 28°C. In each case, first line represents the unloaded biomass biofilm (i.e., control) followed by Cu, Ni, and Pb, respectively.

In order to determine the chemical functional groups/ligands responsible for biosorption processes, FTIR analysis was done using metal-loaded (Cu, Ni, and Pb) and metal-unloaded biomass biofilms of *E. asburiae* ENSD102 (Supplementary Figure 2), *E. ludwigii* ENSH201 (Supplementary Figure 3), *Vitreoscilla* sp. ENSG301 (Figure 8A), *A. lwoffii* ENSG302 (Supplementary Figure 4), and *B. thuringiensis* ENSW401 (Figure 8B). In the present study, several functional groups/ligands including –OH, -NH, –CH, C = O, COO^–^, and P-O were found in different wave numbers (cm^–1^) in metal-unloaded biomass biofilms of different bacterial strains as shown in Figures 8A,B and Supplementary Figures 2–4. Overall, the peak position was shifted in metal-loaded biomass biofilms in response to 100 mg l^–1^ Cu, Ni, and Pb as compared to that of metal-unloaded biomass biofilms.

The IR spectra of metal-unloaded biomass biofilm of *E. asburiae* ENSD102 exhibited C = O of amide groups at 1,641.90 cm^–1^ and were shifted at 1,645.90, 1,636.51 and 1,635.85 cm^–1^ in response to Cu, Ni, and Pb, respectively (Supplementary Figure 2), while COO^–^ of the carboxylate groups appeared at 1,544.10 cm^–1^ in metal-unloaded biomass biofilm of *E. asburiae* ENSD102 and was changed to 1,544.0, 1,545.21, and 1,545.78 cm^–1^. Moreover, phosphate groups and P–O of the (C–PO_2_^–3^) moiety at 1,045 cm^–1^ in metal-unloaded biomass biofilm of *E. asburiae* ENSD102 were shifted to 1,049, 1,083.27, and 1,045.84 cm^–1^ in response to Cu, Ni, and Pb, respectively. Thus, Cu, Ni, and Pb could be sorbed by carbonyl, carboxyl, and phosphate groups of *E. asburiae* ENSD102.

The spectra of the metal-unloaded biomass of *E. ludwigii* ENSH201 exhibited 1,445.90 cm^–1^ for C = O of amide group and were moved to 1,635.98, 1,635.72, and 1,635.99 cm^–1^, respectively, responding to Cu, Ni, and Pb, respectively (Supplementary Figure 3), while 1,544.77 cm^–1^ for COO^–^ of carboxylate group was displaced to 1,544.20, 1,544.36, and 1,546.70 cm^–1^, respectively (Supplementary Figure 2). Furthermore, phosphate group and P–O of the (C–PO_2_^–3^) moiety at 1,045 cm^–1^ in metal-unloaded biomass biofilm were changed to 1,080.50 and 1,047.67 cm^–1^, respectively, challenging to Ni and Pb, respectively. However, phosphate group and P–O of the (C–PO_2_^–3^) moiety at 1,045 cm^–1^ in metal-unloaded and Cu-loaded biomass biofilm were not changed (Supplementary Figure 2). Thus, phosphate groups and P–O of the (C–PO_2_^–3^) moiety of the biomass biofilm of *E. ludwigii* ENSH201 might not be involved in the removal of Cu.

Similarly, shifting of band of metal-unloaded biofilm biomass of *Vitreoscilla* sp. ENSG301 showed a stretched band appearing at 1,645.16 cm^–1^ for C = O of amide groups and was moved to 1,635.28, 1,629.09, and 1,636.07 cm^–1^, respectively, in response to Cu, Ni, and Pb, respectively (Figure 7A), while COO^–^ of the carboxylate groups displayed at 1,545.64 cm^–1^ was changed to 1,545.71, 1,542.37, and 1,545.71 cm^–1^, respectively. Moreover, band positioning at 1,064.70 cm^–1^ was shifted to 1,056.84, 1,053.50, and 1,053.24 cm^–1^ in response to Cu, Ni, and Pb, respectively (Figure 8A). Thus, carbonyl, carboxyl, and phosphate groups of *Vitreoscilla* sp. ENSG301 could sorp Cu, Ni, and Pb.

Compared to the metal-unloaded biomass biofilm of *A. lwoffii* ENSG302, C = O group was remarkably shifted to 10.92, 10.90, and 9.40 cm^–1^ in response to Cu, Ni, and Pb, respectively (Supplementary Figure 3). However, COO^–^ group was moved to 0.66, 1.11, and 0.91 cm^–1^, respectively, responding to Cu, Ni, and Pb, respectively. Band positioning of phosphate groups and P–O of the (C–PO_2_^–3^) moiety were incredibly changed to 30.38, 28.54, and 5.49 cm^–1^, respectively, in response to Cu, Ni, and Pb, respectively (Supplementary Figure 4).

C = O group in metal-unloaded biomass biofilm of *B. thuringiensis* ENSW401 at the position of 1,645.89 cm^–1^ was shifted to 1,636.01, 1,630.25, and 1,636.94 cm^–1^ in responding to Cu, Ni, and Pb, respectively (Figure 8B). The COO^–^ group that appeared at 1,546.68 cm^–1^ in metal-unloaded biomass biofilm was moved to 1,549.05, 1,546.04, and 1,545.85 cm^–1^, challenging to Cu, Ni, and Pb, respectively. Nevertheless, phosphate groups and P–O of the (C–PO_2_^–3^) moiety at 1,084.50 cm^–1^ in metal-unloaded biomass biofilm were shifted to 1,084.38, 1,084.50, and 1,045.32 cm^–1^ in response to Cu, Ni, and Pb, respectively. Thus, carbonyl, carboxyl, and phosphate groups might play a vital role in the biosorption of Cu, Ni, and Pb.

## Discussion

### Environmental Conditions Affect Bacterial Biofilm Formation

In this study, it was observed that environmental cues play a vital role in biofilm formation (Figure 1), production of curli and cellulose (Figures 2–4), and biosorption of Cu, Ni, and Pb (Figures 5–7) by *E. asburiae* ENSD102, *E. ludwigii* ENSH201, *Vitreoscilla* sp. ENSG301, *A. lwoffii* ENSG302, and *B. thuringiensis* ENSW401. *E. asburiae* ENSD102 and *Vitreoscilla* sp. ENSG301 produced the SAL biofilms both at pH 4 (Figure 1E) and pH 8 (Figure 1F), while *E. ludwigii* ENSH201 and *B. thuringiensis* ENSW401 constructed the SAL biofilms only at pH 8 (Figure 1F). However, all these bacterial strains developed the AL biofilm at pH 7 (Figure 1D). Furthermore, only *Vitreoscilla* sp. ENSG301 and *B. thuringiensis* ENSW401, but not other bacterial strains, generated the SAL biofilms in high osmotic condition (Figure 1G). Thus, temperature, pH, and osmolarity and bacterial strains might play an important role in SAL and AL biofilm formation. Numerous researchers have differentiated AL and SAL biofilm genetically, enzymatically, and based on cultural conditions (Friedman and Kolter, 2004; Yap et al., 2005; Haque et al., 2012). Bacterial biofilm formation in different bacterial strains was shown to be influenced by the pH (Hostacká et al., 2010; Ramli et al., 2012; Nguyen et al., 2014; Zhou et al., 2014; Haque et al., 2017), high osmolarity (Lebeer et al., 2007; Hou et al., 2014; Kavamura and de Melo, 2014; Haque et al., 2017), and oxygen tension (Gerstel and Römling, 2001; Bjergbæk et al., 2006; Ahn and Burne, 2007; Liang et al., 2010; Wu et al., 2013). Except temperature (Mosharaf et al., 2018), effect of pH, osmolarity, and oxygen tension on biofilm formation by *E. asburiae* ENSD102, *E. ludwigii* ENSH201, *Vitreoscilla* sp. ENSG301, *A. lwoffii* ENSG302, and *B. thuringiensis* ENSW401 was not studied by any other contemporary researches.

### Bacterial Biofilm Formation as Affected by Nutritional Factors

In ½-strength SOBG media, all these bacterial strains generated thin and fragile AL biofilms (Figure 1H) as compared to those of regular SOBG media (Figure 1D). Shen et al. (2018) reported that at higher nutrient concentrations, biofilms are thicker and denser than under nutrient-poor conditions. Bacterial biofilm formation was also shown to be activated by different divalent cations (such as Mg^2+^ and Ca^2+^) through their effect on electro-static interactions (Patrauchan et al., 2005). Among the divalent cations, Ca^2+^ impacts the mechanical properties of biofilms, as well as cross linkers (Patrauchan et al., 2005). On the other hand, Mg^2+^ increased initial attachment in *P. fluorescens* (Song and Leff, 2006) by reducing the repulsive force between the negatively charged bacterial and substratum surfaces and between negative functional groups of the polysaccharides (Koechler et al., 2015). Haque et al. (2012) have reported that a low concentration (10 μM) of Mg^2+^ increases the AL biofilm formation of *Dickeya dadantii* 3937 in a PhoP-PhoQ-dependent manner. AL biofilm formation controlled by the CytR homolog in *Pectobacterium carotovorum* subsp. *carotovorum* PC1 was also shown to be increased by the different divalent cations, e.g., Mg^2+^, Ca^2+^, Cu^2+^, Zn^2+^, and Mn^2+^ (Haque et al., 2017). In this study, *E. asburiae* ENSD102, *E. ludwigii* ENSH201, *Vitreoscilla* sp. ENSG301, *A. lwoffii* ENSG302, and *B. thuringiensis* ENSW401 also produced the dense and stout AL biofilms by addition of different divalent cations (0.009 M) including Mg^2+^, Ca^2+^, Cu^2+^, and Zn^2+^ (data not shown). Thus, not only environmental cues but also nutritional conditions might play a crucial role in the formation of a thick and robust AL biofilm.

### Environmental and Nutritional Cues Affect Curli and Cellulose Expression in Bacteria

Protein filaments, known as curli, and cellulose played a pivotal role in AL biofilm/pellicle formation in *E. coli* (Prigent-Combaret et al., 2000) and *Salmonella enterica* serovar Enteritidis (White et al., 2003). The production of curli in *Enterobacteriaceae* was shown to be regulated by temperature. For example, curli in *Salmonella* usually is visible under 30°C (Gerstel and Römling, 2003; Bokranz et al., 2005), but some strains such as *S. typhimurium* can express at 37°C (Olsén et al., 1998). Conversely, in clinical isolates of the *E. coli*, the expression of the curli at 37°C is a rarely visible phenomenon (Barnhart and Chapman, 2006). In the present study (in Congo red binding assays), *E. asburiae* ENSD102 and *B. thuringiensis* ENSW401 produced the curli both at 28 and 37°C (Figures 2A,B), while *E. ludwigii* ENSH201 expressed only curli at 28°C (Figure 2A). Curli was not synthesized by *Vitreoscilla* sp. ENSG301 both at 28°C (Figure 2A) and 37°C (Figure 2B), while this bacterial strain generated the curli at pH 4 at 28°C (Figure 2C). The production of cellulose was also found to be dramatically reduced by high temperature (i.e., 37°C), pH 8, high osmolarity, and anaerobic conditions in *E. asburiae* ENSD102, *E. ludwigii* ENSH201, *Vitreoscilla* sp. ENSG301, *A. lwoffii* ENSG302, and *B. thuringiensis* ENSW401 (Figures 3B–D, 4). The expression of the curli fimbriae and cellulose in *Salmonella* spp. is most intense at temperatures under 30°C, in low osmolarity, limited availability of nutrients, and aerobic conditions (Gerstel and Römling, 2003; Solomon et al., 2005; Steenackers et al., 2012). Thus, a variation of biofilm formation in *E. asburiae* ENSD102, *E. ludwigii* ENSH201, *Vitreoscilla* sp. ENSG301, *A. lwoffii* ENSG302, and *B. thuringiensis* ENSW401 in response to environmental and nutritional conditions might be due to differential expression of curli and cellulose.

### Mechanisms of Heavy Metal Tolerance in Bacteria

The determination of MTC in different heavy metals is of particular interest, when bacterial strains are applied for biosorption. In this study, the MTC of Cu, Ni, and Pb was remarkably higher in *B. thuringiensis* ENSW401 as compared to other bacterial strains tested (Table 2). *E. asburiae* ENSD102, *E. ludwigii* ENSH201, *Vitreoscilla* sp. ENSG301, and *A. lwoffii* ENSG302 are Gram-negative bacteria, while *B. thuringiensis* ENSW401 is a Gram-positive bacterium. Generally, the cell wall of both types encompass a peptidoglycan layer that is rich in carboxylate groups and completely surrounds the cell. The peptidoglycan layer of Gram-positive bacterium is thicker (three layers) than the peptidoglycan layer of Gram-negative bacterium (two layers). Thus, cell wall structure might play a pivotal role in MTC of Cu, Ni, and Pb in these bacterial strains. Roane et al. (2009) have reported that (i) binding of metals to extracellular materials of the bacterial cells immobilizes the metals and prevents them from entering into the bacterial cells, (ii) several bacterial strains produced siderophore (iron-complexing, low-molecular-weight organic compounds) complexes that increase metal tolerance, (iii) bacterial strains also generated biosurfactant (excreted from the bacterial cells) complexes of metals that are non-toxic to the cells, and (iv) numerous plasmid-encoded genes [e.g., *cusCBA* (resistance to copper), *cnrCBA* (cobalt–nickel resistance), and *pbrA* (encoding lead resistance)] conferred higher levels of metal tolerance in different bacteria. Thus, several mechanisms might be used in these bacterial strains for the tolerance to Cu, Ni, and Pb.

### Mechanisms of Heavy Metal Toxicity in Bacteria

The growth of *E. asburiae* ENSD102, *E. ludwigii* ENSH201, *Vitreoscilla* sp. ENSG301, *A. lwoffii* ENSG302, and *B. thuringiensis* ENSW401 was severely inhibited with the increase of the concentration of Cu, Ni, and Pb after MTC (data not shown). Bacterial growth, morphological characteristics, and biochemical processes were reported to be disrupted due to toxicity of heavy metals (Roane et al., 2009). Koechler et al. (2015) stated that high concentrations of heavy metals including Cu, Ni, and Pb directly or indirectly generate reactive oxygen species (ROS) upon reacting with DNA, resulting in damaged bases or strand breaks, lipid peroxidation, or protein modification. Roane et al. (2009) reported that metals bind to many cellular ligands and displace essential metals from their native binding sites due to ionic interactions. Moreover, they have shown that metals affect the oxidative phosphorylation and membrane permeability. Some metals can inhibit cellular activity because they present a structural homology with enzyme substrates leading to the metal toxicity. Heavy metal can also cause ion imbalance by adhering to the cell surface and entering through ion channels or transmembrane carriers (Chen et al., 2014). Therefore, future studies should focus on the mechanism of the toxicity of *E. asburiae* ENSD102, *E. ludwigii* ENSH201, *Vitreoscilla* sp. ENSG301, and *B. thuringiensis* ENSW401 in response to higher concentrations of Cu, Ni, and Pb.

### Biofilm Formation in Relation to Heavy Metal Uptake in Bacteria

Biofilm production in *E. asburiae* ENSD102, *Vitreoscilla* sp. ENSG301, and *A. lwoffii* ENSG302 was reported to be affected by 500 to 2,000 mg l^–1^ of CuSO_4_.5H_2_O, Pb(NO_3_)_2_, or NiCl_2_ (Mosharaf et al., 2018). In this study, biofilm production was not remarkably varied in *E. asburiae* ENSD102, *E. ludwigii* ENSH201, *Vitreoscilla* sp. ENSG301, *A. lwoffii* ENSG302, and *B. thuringiensis* ENSW401 in response to 12.5 to 200 mg l^–1^ Cu, Ni, or Pb (Supplementary Figure 1). Accordingly, biosorption capacity was not significantly varied in these bacterial strains in response to 12.5, 25, and 50 mg l^–1^ Cu, Ni, and Pb (Figure 5). Interestingly, only *Vitreoscilla* sp. ENSG301 and *B. thuringiensis* ENSW401 completely removed (100%) Cu and Ni at an initial concentration of 12.5 mg l^–1^, while all these bacterial strains totally removed (100%) Pb at initial concentrations of 12.5 and 25 mg l^–1^ at pH 7 and 28°C. However, *Vitreoscilla* sp. ENSG301 and *B. thuringiensis* ENSW401 removed much more Cu, Ni, or Pb as compared to *E. asburiae* ENSD102, *E. ludwigii* ENSH201, and *A. lwoffii* ENSG302 in response to 100, 150, and 200 mg l^–1^ (Figure 5). Thus, biosorption might be dependent on both concentrations of the heavy metals (Cu, Ni, and Pb) and bacterial strains. At present, various ordinance, laws, rules, acts, and policies have been made to control environmental pollution in Bangladesh. The Department of Environment of Bangladesh also set safety limit of different heavy metals in industrial effluent [DoE (Department of Environment), 2008]. The World Health Organization [WHO (World Health Organization), 2017], European Union [European Union (EU), 2002], United States Environmental Protection Agency [USEPA (United States Environmental Protection Agency), 2012], and Bangladesh [DoE (Department of Environment), 2008] prescribed the maximum acceptable concentrations at (mg l^–1^) 2.0, 0.5, 0.2, and 0.5, respectively, for Cu; 0.02, 0.5, 0.2, and 1.0, respectively, for Ni; and 0.01, 0.5, 0.05, and 0.10, respectively, for Pb. Thus, treated wastewater by these bacterial strains at an initial concentration of 12.5 mg l^–1^ for Cu and 12.5 and 25.0 mg l^–1^ for both Ni and Pb are within the safety limit set by the abovementioned organizations, while concentrations of Cu, Ni, and Pb are higher than the maximum acceptable values in all other treated samples.

### Cellular Structure, EPS, and Enzymes on Metal Biosorption in Bacteria

Bacterial cellular structure, EPS, and extracellular enzyme play a vital role in metal biosorption. Both Gram-positive and Gram-negative bacterial cell wall contain peptidoglycan that determines the metal-binding capability. However, teichoic acids are present only in Gram-positive bacteria that provide an overall negative charge to the cell wall, due to the presence of phosphodiester bonds. On the other hand, lipopolysaccharides (LPS) are only present in Gram-negative bacteria that confer an overall negative charge to the cell wall of Gram-negative bacteria. The anionic functional groups present in the cell wall of Gram-positive and Gram-negative bacteria are the key contents primarily responsible for the anionic character and metal-binding or biosorption capacity of the cell wall (Moat et al., 2002). Notably, bacterial biofilm EPS also play a key role in metal biosorption (Pal and Paul, 2008; Li and Yu, 2014). Among the contents of the EPS, proteins form complexes with heavy metal ions (Mejáre and Bülow, 2001), while polysaccharides cross-link with metals (Li and Yu, 2014). Biofilm EPS matrix has abundant chemical functional groups such as amino, carboxyl, carboxylate, phosphate, and hydroxyl groups (Mosharaf et al., 2018). It was reported that negatively charged functional groups present in the EPS matrix formed organometallic complexes with multivalent metal cations via electrostatic interactions and subsequent metal removal (Gutnick and Bach, 2000). Numerous extracellular enzymes (e.g., protease, peptidase, endo-cellulase, α-glucosidase, β-glucosidase, peroxidase, etc.) have been detected in bacterial biofilms (Flemming and Wingender, 2010). Many of them were reported to degrade the contents of EPS (Flemming and Wingender, 2010) and detoxify the heavy metals (Pal and Paul, 2008). However, extracellular enzymes synthesized by *E. asburiae* ENSD102, *E. ludwigii* ENSH201, *Vitreoscilla* sp. ENSG301, and *B. thuringiensis* ENSW401 and their involvement in detoxification of heavy metals are yet to be examined.

### Physicochemical Conditions Alter Metal Biosorption in Bacteria

Removal of heavy metals from aqueous solution by bacteria is a complex process due to the effect of different physicochemical factors such as initial metal concentration, temperature, pH, time, ionic strength, and metal chemistry (Gabr et al., 2008; Hassan et al., 2009). In this study, increasing the concentrations of Cu, Ni, and Pb decreased the metal biosorption (Figure 5). Higher metal biosorption at lower concentrations of heavy metals was reported to be due to the availability of free metal-binding sites, while lower metal biosorption at higher concentrations is due to lack of free metal-binding sites (Kaduková and Virèíková, 2005; Oves et al., 2013; Kirova et al., 2015).

Cellulose-based materials including cellulose gels, cellulose composites, cellulose derivatives, functionalized cellulose, and nano-crystaline cellulose are widely used for the adsorption of heavy metals from wastewater (Jamshaid et al., 2017). *E. asburiae* ENSD102, *E. ludwigii* ENSH201, *Vitreoscilla* sp. ENSG301, *A. lwoffii* ENSG302, and *B. thuringiensis* ENSW401 were shown to produce nanocellulose that is amorphous in nature (Mosharaf et al., 2018). In this study, cellulose production (Figure 4) and biosorption of Cu, Ni, and Pb by these bacterial strains were dramatically reduced at 37°C as compared with 28°C (Figure 6). Bacterial cellulose production was shown to be linked with biosorption of metals (Teitzel and Parsek, 2003; Li and Yu, 2014). Thus, reduction of the removal of Cu, Ni, and Pb by these bacterial strains at 37°C might be due to a lower production of cellulose. Redha (2020) has shown that metal biosorption is not highly affected in temperatures ranging from 20 to 35°C. On the other hand, Tas̨ar et al. (2014) have reported that increaseing temperature from 20 to 40°C decreases the surface activity of biosorbents such as peanut shells leading to decreased biosorption of Pb.

In this study, pH levels regulated the biosorption of Cu, Ni, and Pb (Figure 7). The difference in metal biosorption at different pH was associated with the effect of both the chemistry of the functional groups and the chemistry of metal ions (Wei et al., 2016). At low pH, functional groups present in the biofilm EPS tightly bound with hydronium ions leading to restricting the binding of metal cations due to repulsive force. Conversely, with increasing pH, various functional groups including carbonyl, carboxyl, phosphate, and amino start experiencing negative charges due to deprotonation leading to binding with metal cations and thus increasing the biosorption capacity (Oves et al., 2013; Abdi and Kazemi, 2015). In this study, the chemical functional groups present in the biofilm EPS of *E. asburiae* ENSD102, *E. ludwigii* ENSH201, *Vitreoscilla* sp. ENSG301, *A. lwoffii* ENSG302, and *B. thuringiensis* ENSW401 were determined using FTIR in both metal-loaded and -unloaded (control) samples at an initial Cu, Ni, and Pb concentration of 100 mg l^–1^, pH 7, and at 28°C. Several functional groups including –OH, –NH, –CH, C = O, COO-, and P-O were detected in the samples (Figures 8A,B and Supplementary Figures 2–4). FTIR results revealed that Cu, Ni, and Pb could be sorbed by carbonyl, carboxyl, and phosphate groups of *E. asburiae* ENSD102, *Vitreoscilla* sp. ENSG301, *A. lwoffii* ENSG302, and *B. thuringiensis* ENSW401, while phosphate groups and P–O of the (C–PO_2_^–3^) moiety have no role in Cu removal by *E. ludwigii* ENSH201. The chemical functional groups in metal-loaded biofilm EPS in *E. asburiae* ENSD102, *E. ludwigii* ENSH201, *Vitreoscilla* sp. ENSG301, *A. lwoffii* ENSG302, and *B. thuringiensis* ENSW401 were not reported by any other contemporary researches.

### Future Perspective and Scale Up

Future studies should focus on the biosorption of heavy metal from real wastewater by these bacterial strains. The mechanisms involved in metal toxicity in these bacterial strains should be studied. Extracellular enzymes produced in the EPS matrix of these bacterial strains and their role in the detoxification of heavy metals should also be examined. Nevertheless, genetic engineering tools should be used to construct the engineered *E. asburiae* ENSD102, *E. ludwigii* ENSH201, *Vitreoscilla* sp. ENSG301, *A. lwoffii* ENSG302, and *B. thuringiensis* ENSW401 with higher metal sorption capacity. For scale up, large amounts of biomass biofilm can be produced in less expensive growth media by using these bacterial strains. Currently, bacterial biofilm biomass is being used in different types of bioreactors including fixed bed reactor, packed bed reactor, and fluidized bed reactor to remove heavy metals from wastewater. Thus, biomass biofilm produced by *E. asburiae* ENSD102, *Vitreoscilla* sp. ENSG301, *A. lwoffii* ENSG302, and *B. thuringiensis* ENSW401 can be utilized in the *ex situ* conditions for different engineered bioreactor systems. This will require an interdisciplinary approach with the integration of metallurgical, chemical, mathematical, and civil engineering skills along with sorption and wastewater treatment plan to combat heavy metal pollution from the aquatic environment. The last but not the least, in order to get the best out of the results obtained and to get this technology used for heavy metal pollution mitigation, it needs to be integrated into the policy simultaneously by the concerned government and international donor agencies.

## Conclusion

Biofilm formation and biofilm matrix compounds such as curli and cellulose production in *E. asburiae* ENSD102, *E. ludwigii* ENSH201, *Vitreoscilla* sp. ENSG301, *A. lwoffii* ENSG302, and *B. thuringiensis* ENSW401 were remarkably affected by different environmental and nutritional conditions. Only *Vitreoscilla* sp. ENSG301 and *B. thuringiensis* ENSW401 completely removed (100%) Cu and Ni at an initial concentration of 12.5 mg l^–1^, while all these strains totally removed (100%) Pb at initial concentrations of 12.5 and 25 mg l^–1^ at pH 7 and 28°C. FTIR study showed that Cu, Ni, and Pb could be sorbed by carbonyl, carboxyl, and phosphate groups of the biomass biofilms of *E. asburiae* ENSD102, *Vitreoscilla* sp. ENSG301, *A. lwoffii* ENSG302, and *B. thuringiensis* ENSW401, while phosphate groups and P–O of the (C–PO_2_^–3^) moiety have no role in Cu removal by *E. ludwigii* ENSH201. Thus, all these bacterial strains can be utilized in biosorption of heavy metals from wastewater.

## Data Availability Statement

The original contributions presented in the study are included in the article/Supplementary Material, further inquiries can be directed to the corresponding author.

## Author Contributions

MMH conceived the idea, developed the methodologies, conducted the experiments, wrote the manuscript, and collected the research fund. MKM and MZHT conducted the experiments. MAH and MKA performed FTIR and AAS, respectively. All authors read the manuscript and approved for publication.

## Conflict of Interest

The authors declare that the research was conducted in the absence of any commercial or financial relationships that could be construed as a potential conflict of interest.
