# Supplementary figures and images for: Biofilm Formation, Production of Matrix Compounds and Biosorption of Copper, Nickel and Lead by Different Bacterial Strains
Source: Front Microbiol. 2021 Jun 10;12:615113. doi: 10.3389/fmicb.2021.615113 (PMC8222582; doi:10.3389/fmicb.2021.615113)

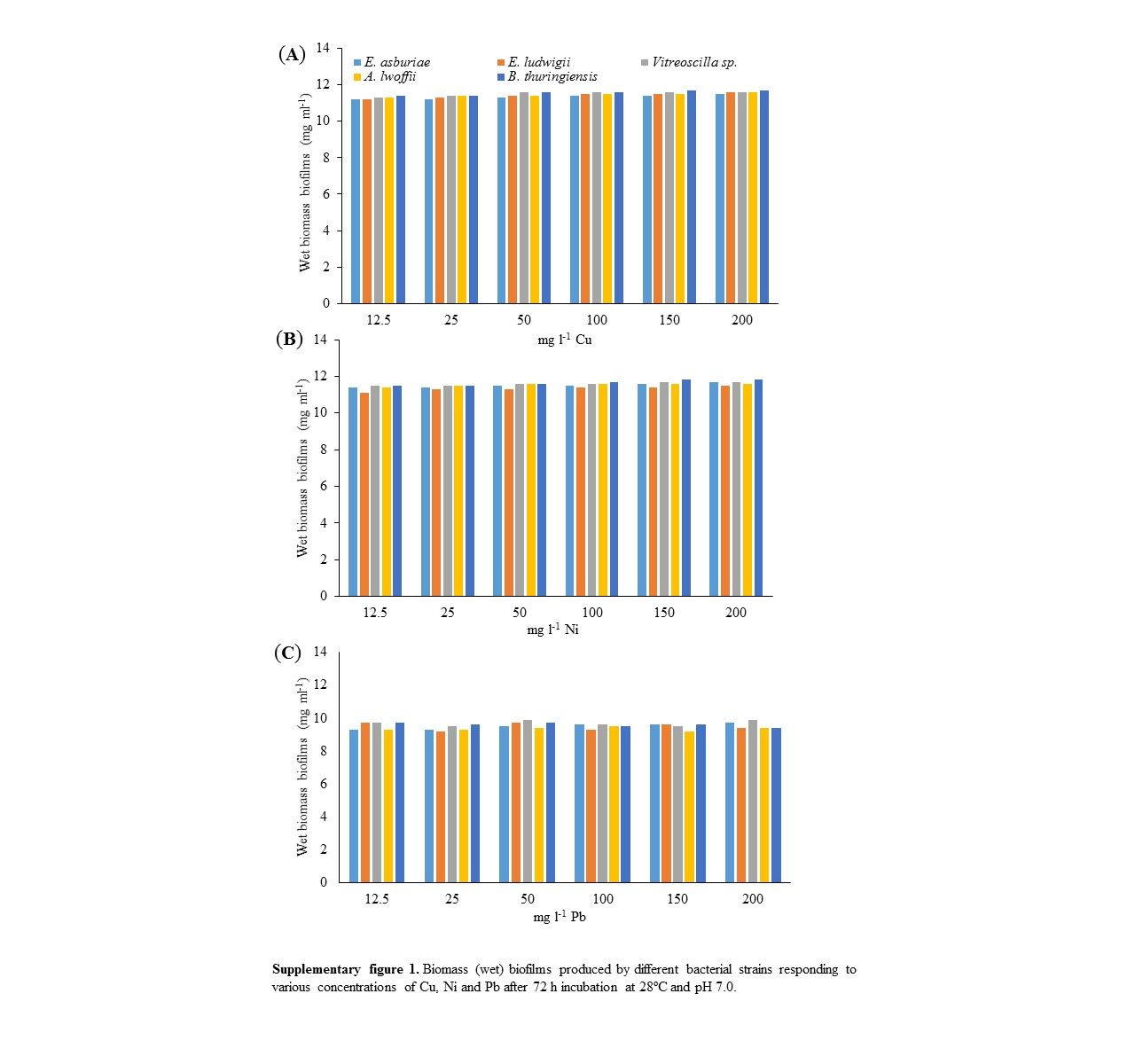

Supplement: Supplementary file 1 [file Image_1.jpg]

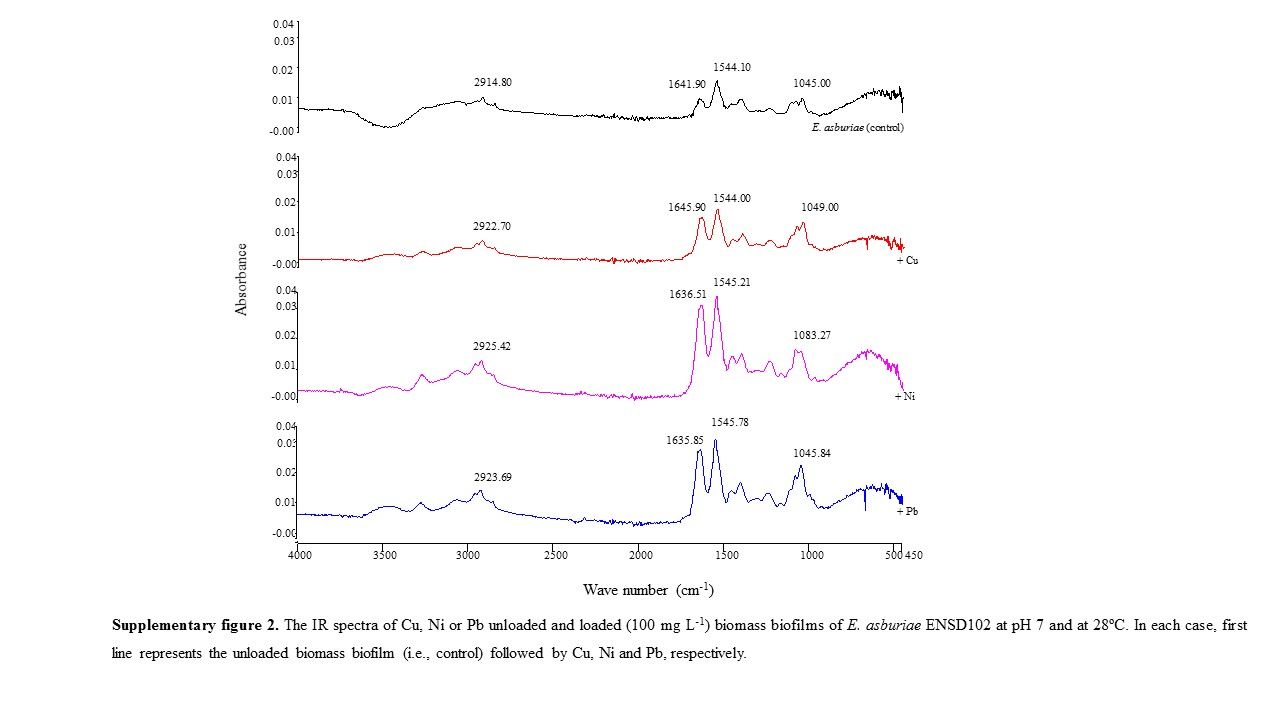

Supplement: Supplementary file 2 [file Image_2.JPEG]

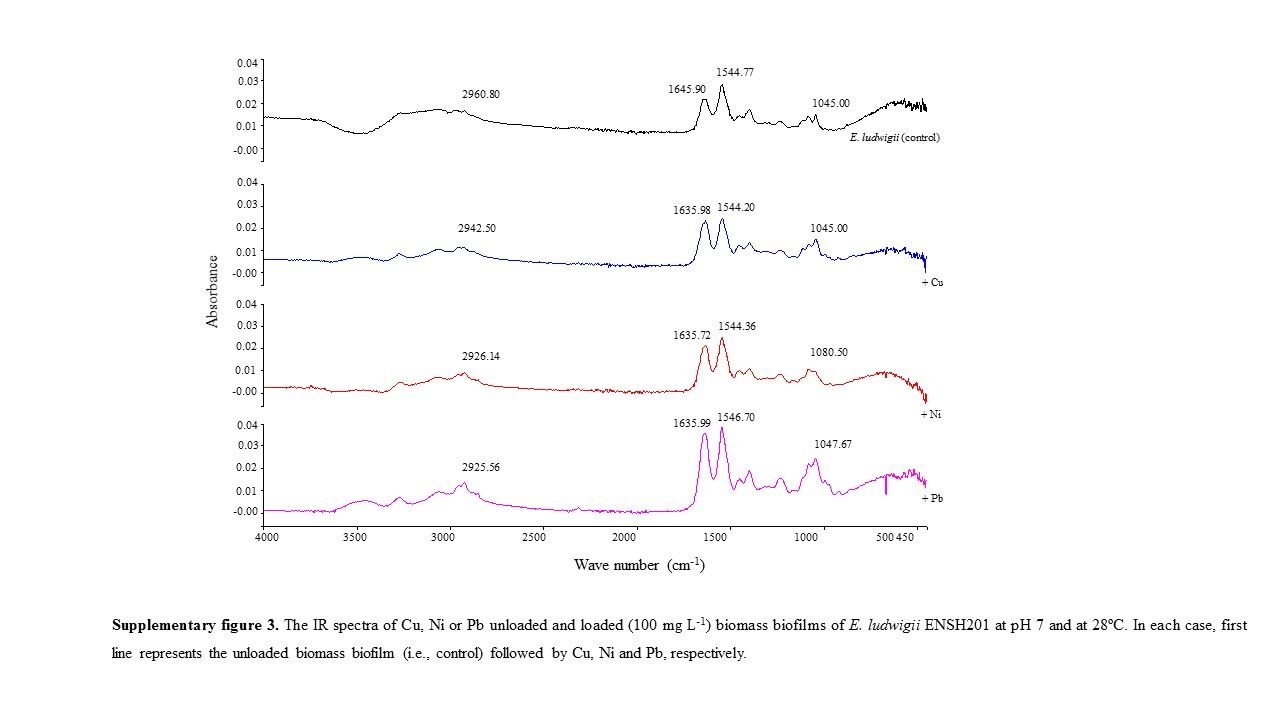

Supplement: Supplementary file 3 [file Image_3.JPEG]

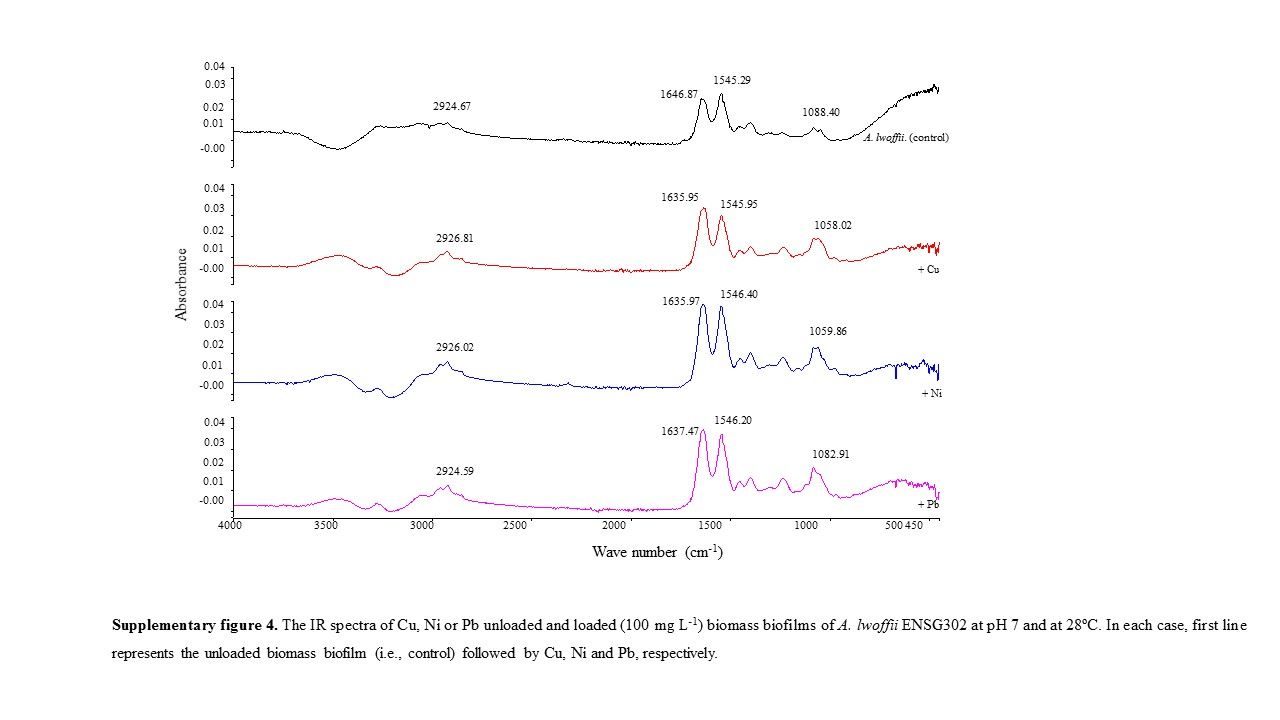

Supplement: Supplementary file 4 [file Image_4.jpg]
